# P-1817. High-Throughput Neutralizing Antibody (nAb) Assay Using a Pseudovirus Platform for Seasonal and Pandemic Influenza Studies

**DOI:** 10.1093/ofid/ofaf695.1986

**Published:** 2026-01-11

**Authors:** Kai Sha, Samuel Jauregui, Christos J Petropoulos, Terri Wrin

**Affiliations:** LabCorp-Monogram Biosciences, South San Francisco, CA; LabCorp-Monogram Biosciences, South San Francisco, CA; Monogram Biosciences, San Francisco, California; Labcorp-Monogram, South San Francisco, California

## Abstract

**Background:**

In early 2024, a high-pathogenicity avian influenza (HPAI) H5N1 began spreading from wild birds to domestic fowl and eventually livestock and wildlife in the United States. Human infections have been limited to individuals exposed to infected animals. Concurrently seasonal viruses (H1N1, H3N2, B) are circulating at high levels causing significant morbidity.

A high-throughput neutralizing antibody (nAb) assay platform has been developed that is capable of conducting 6000 assays per week and can be utilized for in-depth and widespread surveillance of natural infections, as well as the evaluation of seasonal and pandemic influenza vaccine responses. This assay employs a well-established pseudovirus assay platform that limits virus replication to a single cycle. As a result, it enables the evaluation of nAb responses to highly contagious and/or pathogenic influenza strains, such as the H1N1, H3N2, B strains and H5N1, H7N9.

**Figure:**
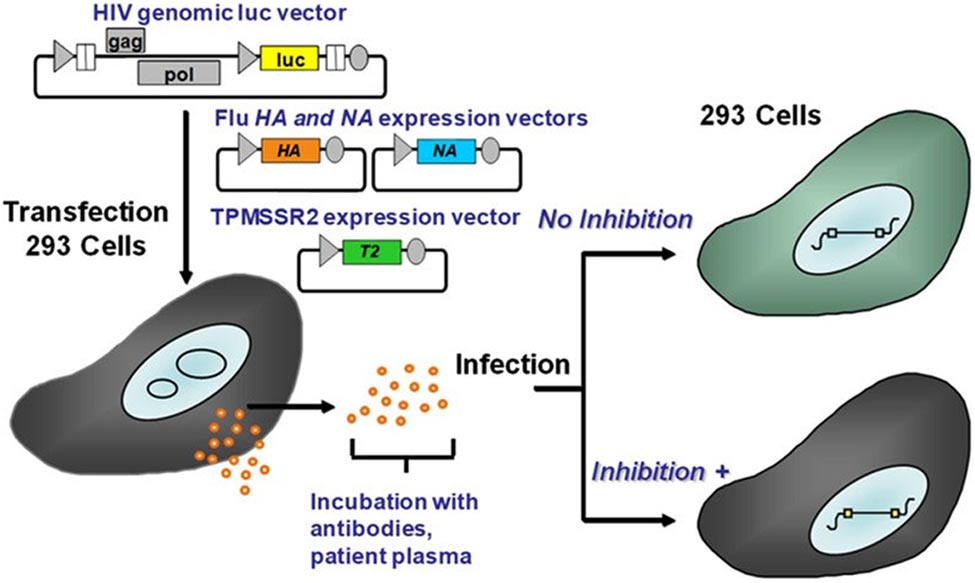
Illustration of influenza neutralizing antibody (nAb) assay

**Figure:**
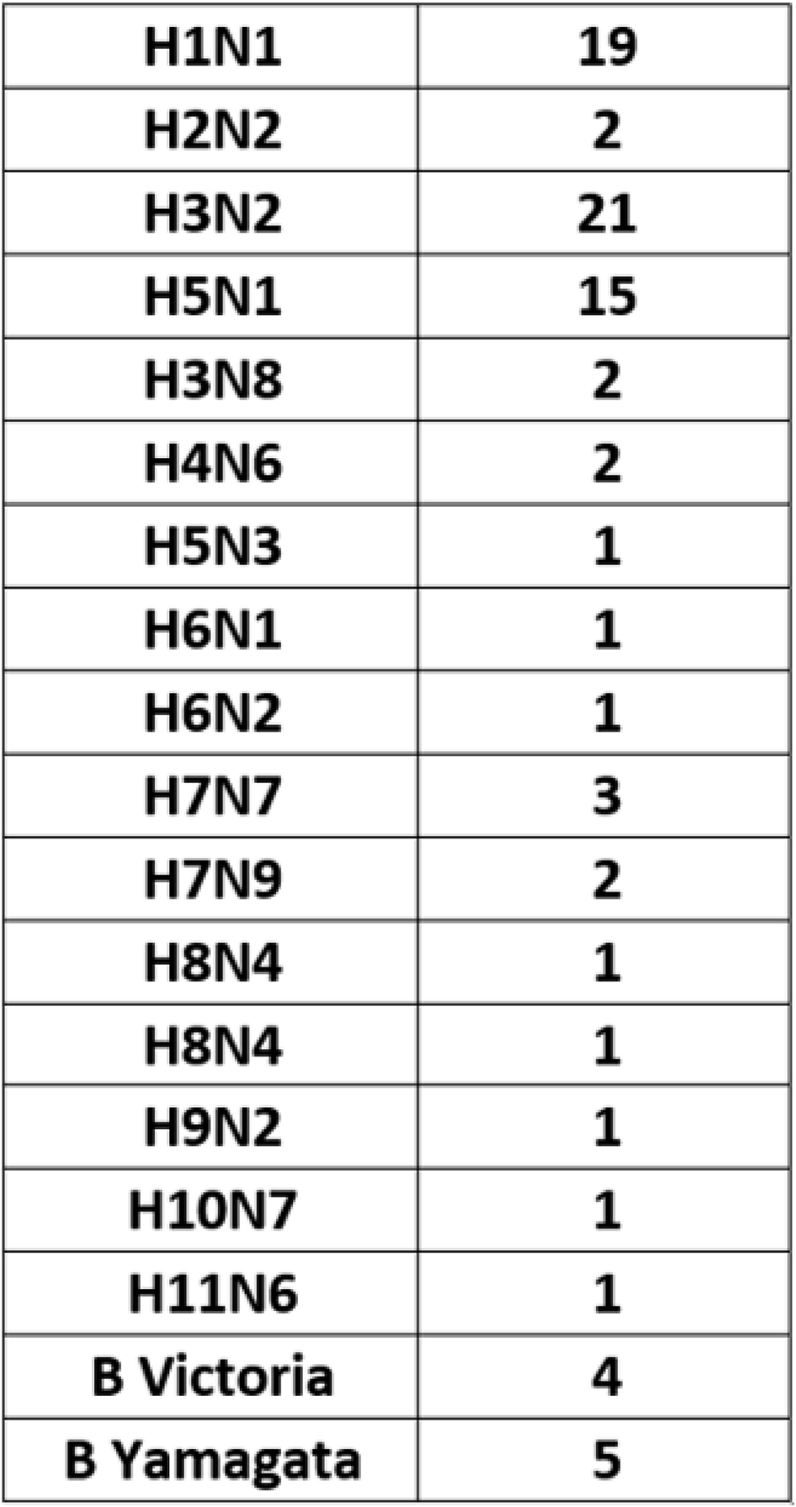
Comprehensive panel of influenza pseudovirus with number of strains per subtype.

**Methods:**

HIV pseudovirus stocks that carry a luciferase reporter gene and express influenza HA and NA proteins are generated and incubated with serial dilutions of sera/plasma, mAb or purified antibodies, followed by inoculation of HEK293 target cells (Fig. 1). Pseudovirus infection that is not neutralized results in luciferase production, while neutralization reduces or prevents luciferase production.

**Figure:**
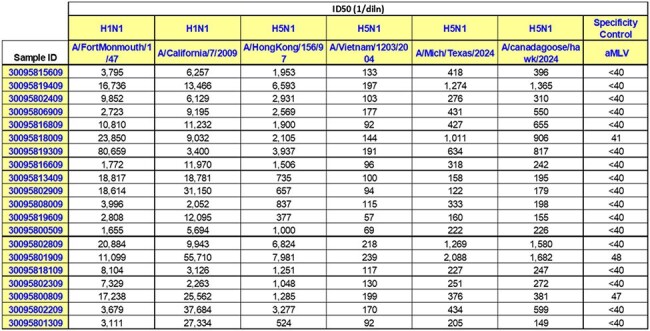
Neutralization of H1N1 and H5N1 pseudoviruses by a panel of randomly selected human sera.

**Figure:**
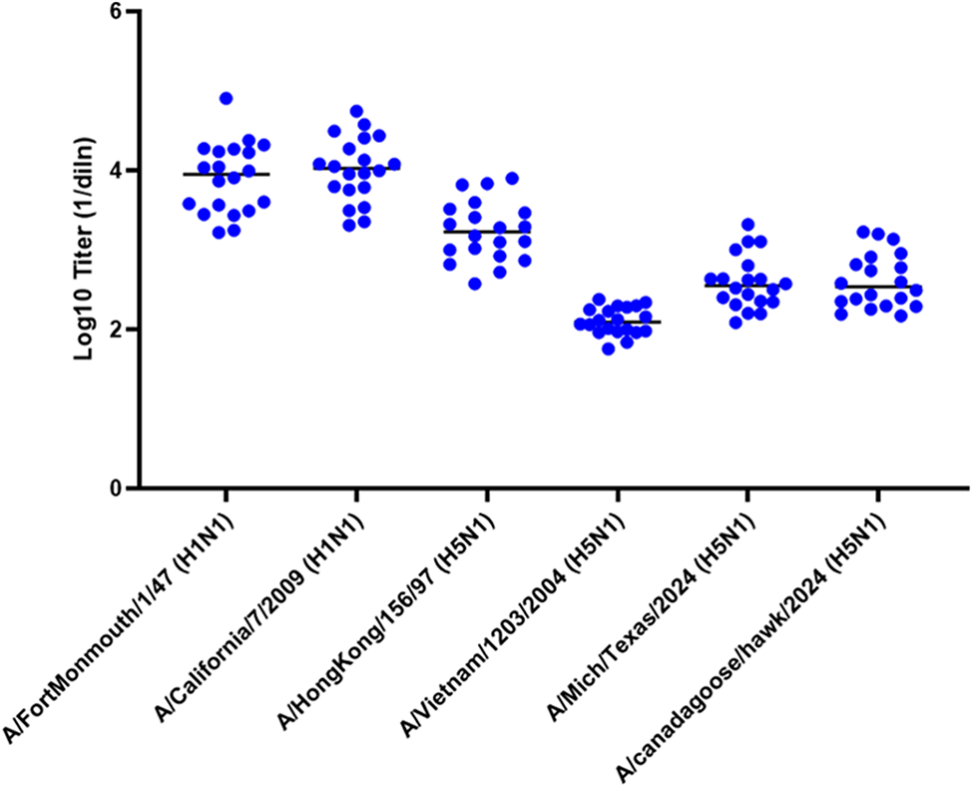
Neutralization antibody titers from Table 2

**Results:**

A comprehensive panel of pseudovirus stocks has been assembled (Table 1). The panel includes H5N1 vaccine strains, human and avian H5N1 field strains, seasonal influenza strains such as H3N2, H1N1 and influenza B. Testing has been conducted using a broad panel of animal antisera and mAb against multiple H5N1 and H1N1 strains demonstrating strain-specific responses with limited cross-reactivity. Interrogation of a panel of 20 human sera collected from 2020-2024 revealed measurable nAb titers directed against historical and seasonal strains (Table 2 and Fig. 2). However, cross neutralization of 2024 H5N1 human and avian isolates were also observed.

**Conclusion:**

Sera of humans previously exposed to seasonal influenza and/or immunization possess a limited ability to neutralize avian H5N1. The impact of low titer H5N1 responses on zoonotic events leading to widespread human to human transmission is uncertain.

**Disclosures:**

Christos J. Petropoulos, PhD, Labcorp-Monogram Biosciencs: employee|Labcorp-Monogram Biosciencs: Stocks/Bonds (Public Company) Terri Wrin, PhD, Labcorp (Monogram Biosciences): Stocks/Bonds (Public Company)

